# Mechanisms of Lipid‐Associated Macrophage Accrual in Metabolically Stressed Adipose Tissue

**DOI:** 10.1002/bies.202400203

**Published:** 2025-01-19

**Authors:** Isabel Reinisch, Sarah Enzenhofer, Andreas Prokesch

**Affiliations:** ^1^ Department of Health Sciences and Technology, Institute of Food Nutrition and Health Eidgenössische Technische Hochschule Zürich (ETH) Schwerzenbach Switzerland; ^2^ Gottfried Schatz Research Center for Cell Signaling Metabolism and Aging, Division of Cell Biology, Histology and Embryology Medical University of Graz Graz Austria; ^3^ BioTechMed‐Graz Graz Austria

**Keywords:** adipose, lipid‐associated macrophages, metabolic syndrome, metaflammation, obesity

## Abstract

Adipose tissue (AT) inflammation, a hallmark of the metabolic syndrome, is triggered by overburdened adipocytes sending out immune cell recruitment signals during obesity development. An AT immune landscape persistent throughout weight loss and regain constitutes an immune‐obesogenic memory that hinders long‐term weight loss management. Lipid‐associated macrophages (LAMs) are emerging as major players in diseased, inflamed metabolic tissues and may be key contributors to an obesogenic memory in AT. Our previous study found that LAM abundance increases with weight loss via intermittent fasting (IF) in obese mice, which is driven by adipocyte p53 signalling. However, the specific signals causing LAM accumulation in AT under IF remain unknown. In this piece, we hypothesise on a range of adipocyte‐secreted signals that can harbor immune‐attractive features upon fasting/refeeding cycles. We highlight possible mechanisms including cell death signalling, matrikines, and other damage‐associated molecular patterns (DAMPs), as well as adipo(‐cyto)kines, lipid mediators, metabolites, extracellular vesicles, and epigenetic rewiring. Finally, we consider how advances in mechanisms of AT LAM recruitment gleaned from preclinical models might be translatable to long‐term weight management in humans. Thus, we provide vantage points to study signals driving monocyte recruitment, polarisation towards LAMs, and LAM retention, to harness the therapeutic potential of modulating AT LAM levels by impacting the immune‐obesogenic memory in metabolic disease.

## Graphical abstract

Intermittent fasting (IF) exacerbates lipid‐associated macrophage (LAM) accrual in adipose tissue of obese mice via an adipocyte p53‐dependent mechanism. Possible adipocyte‐derived, IF‐mediated signals for monocyte recruitment and macrophage differentiation in the context of weight cycling include apoptotic or lipid/metabolite signals, DAMPs, matrikines, adipo(cyto)kines, extracellular vesicles, and epigenetic rewiring.2

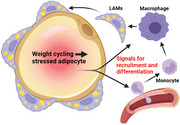

## 1 Introduction

### 1.1 The Metabolic Syndrome, Weight Loss, and Weight Regain

Obesity continues to be a global problem with rising incidence and a host of co‐morbidities. These include adiposity, local and systemic insulin resistance, and metabolic‐associated steatotic liver disease (MASLD), together amounting to the pathologic umbrella term known as metabolic syndrome (MetSyn) [1, 2]. Adipose tissue (AT) inflammation, characterised by immune cell infiltration into metabolically stressed AT, is considered a key indicator of MetSyn [3] and linked to the exacerbation of dysfunctional metabolism, such as insulin resistance or dyslipidemia [4]. Measures to curtail obesity‐related MetSyn include lifestyle interventions (dietary restriction, like fasting, and exercise [5, 6, 7]), surgical approaches (e.g., bariatric surgery) [8], and, since recently, pharmacological means such as incretin analogues [9]. While these interventions can lead to effective weight loss and amelioration of MetSyn hallmarks, there is still a strong demand for research on sustainable weight loss and long‐term weight management. This is supported by ample evidence of rapid weight regain after cessation of fasting or pharmacological treatment protocols, aggregating to a regain of 80% of the lost weight within 5 years, even in a subgroup of bariatric surgery patients [10, 11]. Repetitive weight loss and regain (prominent in intermittent fasting [IF] or yo‐yo dieting) may exacerbate symptoms of the MetSyn such as insulin resistance and liver steatosis [10, 11]. This argues for the existence of an obesogenic memory that stores information about prior metabolic dysfunction throughout weight cycling.

### 1.2 Metaflammation in Adipose Tissue: A Case for an Immune‐Obesogenic Memory

While the microbiome and central nervous system are proposed regulators of an obesogenic memory, retained metaflammation in AT could comprise another central mechanism [12]. Metaflammation denotes a chronic, low‐grade inflammatory state of tissues important for metabolic homeostasis and is recognissed as a strong contributor to the MetSyn [13]. Among the leucocytes that infiltrate metabolic tissues, monocyte‐derived macrophages are prominent especially in AT of obese individuals [14, 15, 16]. Metabolic disease–associated macrophages are distinct from tissue‐resident macrophages and come in many different flavors [16, 17]. Sequencing‐based single‐cell analyses of AT and liver established lipid‐associated macrophages (LAMs) as a prevalent subtype of metabolic disease–related macrophages in mice and humans [18]. Recent studies revealed that during and after weight loss in obese mice, metaflammation persists and leads to increased weight regain and exaggerated MetSyn symptoms [19, 20]. Compositional shifts included compromised recovery of Type 2 regulatory cells, enhanced activation of antigen‐presenting cells, CD8^+^ T‐cell exhaustion, and an accumulation of macrophages. In particular, one study demonstrated retention of AT LAMs after weight loss in formerly obese mice, with their numbers increasing significantly upon weight regain [19], which could implicate LAMs in the development of an immune‐obesogenic memory. We could recently show that LAMs are a predominant macrophage subtype in visceral AT of obese mice and that their abundance is strongly increased after an IF regimen, comprised of nine cycles of 24‐h water‐only fasting and ad libitum high‐fat diet (HFD) refeeding over 3 weeks [21]. Although the cumulative food intake with this protocol is similar between non‐fasting and fasting groups, IF led to cycles of weight loss and weight regain resulting in significant net weight loss at the end of the protocol. Strikingly, IF‐mediated LAM accrual was blunted when p53 knockout was initiated in adipocytes before IF [21]. Furthermore, ablation of p53 in adipocytes increased the response to IF (higher ketone bodies and weight loss), amplified the metabolic benefits of IF in mice, and p53 status was linked to the efficacy of weight loss interventions in humans. p53 is a major tumour suppressor but also functions in cancer‐unrelated ways to regulate local and systemic metabolic homeostasis [22, 23, 24]. Data on p53 signalling as an interface between parenchymal and immune cells is, however, rare and knowledge about the regulation of LAM abundance in AT is largely missing. Mechanistically, p53‐mediated LAM accrual may limit the effectiveness of weight loss interventions by affecting the sensitivity of adipocytes to catabolic stimuli, a process that was previously linked to inflammation‐induced adipocyte dysfunction [25]. This hypothesis is supported by elevated plasma‐free fatty acids and increased expression of β‐adrenergic receptor 3 as well as genes of the entire lipolytic cascade in adipocytes upon p53 knockout [21]. If and how LAMs contribute to an immune‐obesogenic memory remains enigmatic, due to the lack of LAM depletion experiments in weight cycling scenarios. As one possibility, adipocytes and LAMs might engage in futile lipid cycling [26] instead of providing fatty acids as substrate for liver ketogenesis. Upon cessation of IF, the fatty acids buffered in LAMs might, therefore, be readily available for re‐esterification into adipocyte triglycerides, which could cause exacerbated adipocyte hypertrophic growth and accelerated weight regain. As a corollary, therapeutic strategies targeting LAMs [27] may impinge on the above mechanisms to improve weight loss‐efficiency and/or long‐term weight management. However, several points need to be considered before embarking on LAM‐based therapies. Firstly, LAMs are very plastic and can adopt both a pro‐ or anti‐inflammatory phenotype, in a tissue and context‐dependent manner [28]. Thus, the mechanisms that control LAM heterogeneity need to be carefully investigated. Secondly, reducing the number of LAMs in AT of obese mice, without addressing the upstream recruitment signalling, has been found to exacerbate AT dysfunction and worsen metabolic health [29, 30]. This data indicates that the therapeutic potential of LAMs can only be fully realised by also addressing the initial stimulus that attracts LAMs.

In this piece, we hypothesise possible mechanisms leading to increased LAM populations in AT upon weight cycling.

## 2 Potential Mechanisms of LAM Recruitment to AT

**FIGURE 1:**
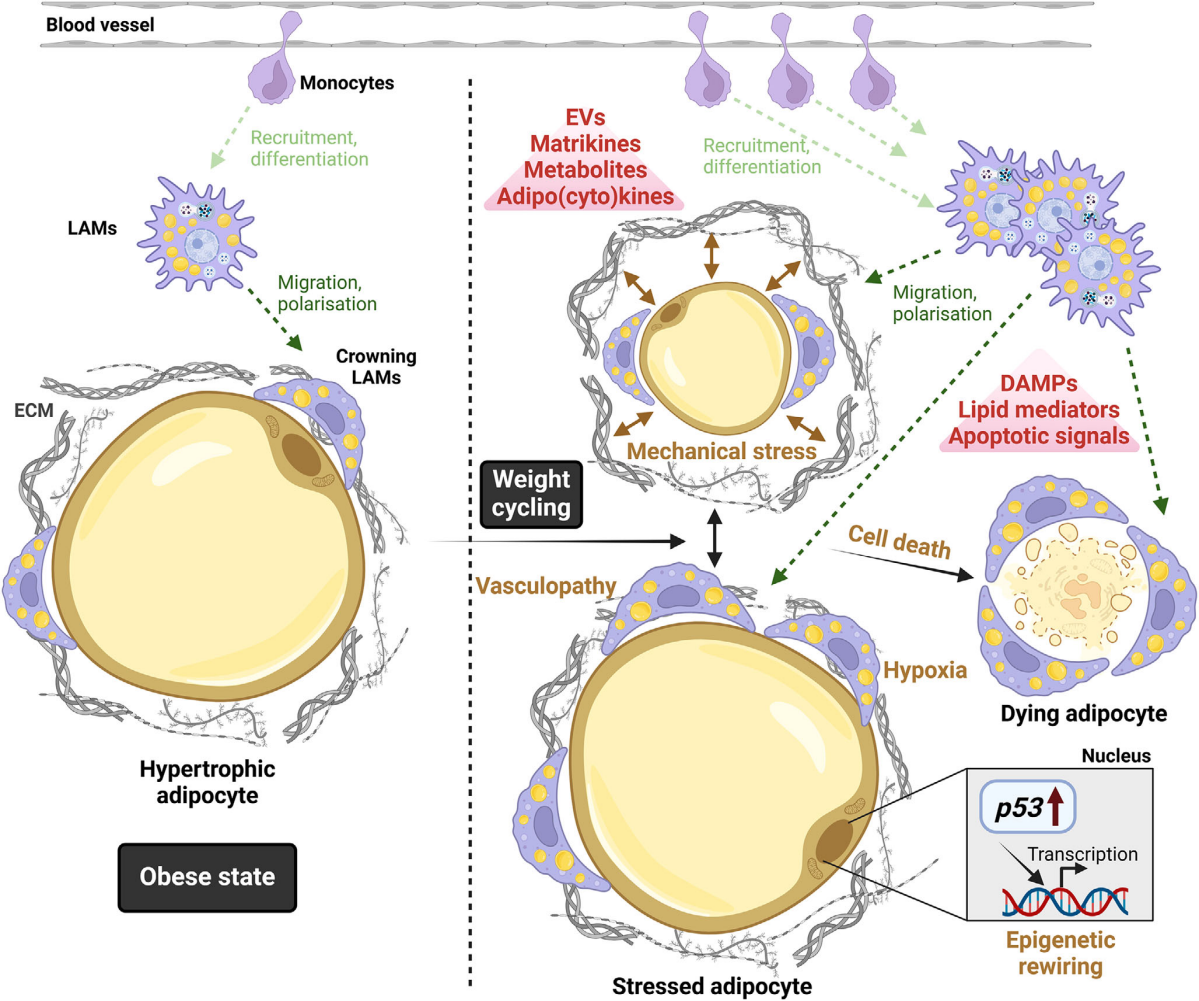
Summary of proposed LAM recruitment mechanisms in AT upon weight cycling. Growing and shrinking of adipocytes upon weight cycling causes cellular stresses (in brown) that can culminate in cell death and lead to the transmission of stress signals (in red) to recruit monocytes and regulate LAM differentiation and polarisation. DAMPs, damage‐associated molecular patterns; ECM, extracellular matrix; EVs, extracellular vesicles; LAMs, lipid‐associated macrophages. Created with BioRender.com.

During sterile inflammation, the increase in the abundance of infiltrating immune cells is generally dictated by immuno‐attractive, chemotactic signals emanating from stressed or dying cells [31, 32, 33]. In the context of tissues involved in metabolic homeostasis, these signals are received from circulating monocytes that follow gradients to ultimately extravasate, migrate towards the signal maximum (i.e., the site of stress), and differentiate into their phagocytic effector states [33]. While the local proliferation of macrophages can increase their abundance in AT [34], we focus on signals that elicit recruitment and differentiation from circulating monocyte pools, as this seems to be the predominant mechanism for LAM accumulation in metabolically challenged tissues [18]. Therefore, we discuss general immuno‐attractive processes and chemotactic signals that appear as plausible players in the adipocyte‐immune axis in metabolically challenged AT (summarissed in Figure 1), especially as informed by our diet‐induced obese mouse model with IF [21].

### 2.1 Adipocyte Death: Apoptotic Bodies, Lipid Messengers, and Find‐Me Signals

Steady growth of AT depots during the development of obesity entails adipocyte hypertrophy and fibrosis, along with poor vascularization, which leads to nutrient and oxygen undersupply. All these stressors, especially if occurring together, can culminate in adipocyte cell death, which can emit immuno‐attractive “find‐me” signals to facilitate the clearance of apoptotic cells and buffering of liberated lipids [31, 32]. As the membranes of apoptotic cells stay intact, they release “find‐me” signals either in soluble form or incorporated in apoptotic bodies. These signals include (membrane) lipids such as lysophosphatidic acid and sphingosine‐1‐phosphate (S1P), chemokines (such as CCL2 and CX3CL1), and nucleotides (mainly ATP and UTP) [33]. In turn, they act on receptors of phagocytic cells or their precursors. As LAMs have a strong capacity to efferocytose [18], it is very likely that apoptotic “find‐me” signals contribute to monocyte recruitment and LAM differentiation as shown for liver disease [35], although we lack direct evidence from metabolically stressed AT. Additionally, the cellular origin of apoptotic debris that macrophages engulf determines their cellular identity and may be responsible for macrophage plasticity, leading to the formation of various subclasses [36]. Our recent work suggests that apoptosis is activated in visceral AT upon IF in a diet‐induced obese mouse model [21]. This increase in apoptotic signalling in adipocytes coincides with massive AT LAM accrual. Blunting of this LAM accrual upon adipocyte‐specific knockout of p53, a major apoptotic regulator, indeed indicates that apoptosis may play an important role in AT LAM recruitment, at least in our experimental setting [21].

In general, apoptotic signalling might impact local macrophage populations to elicit a LAM phenotype. However, if the reach of apoptotic signals in space and time (as determined by their half‐life) is sufficient to attract monocytes from circulation remains to be investigated. Further studies also need to delineate if single apoptotic messengers like S1Ps are sufficient to recruit LAMs in AT or if concerted action of several mediators is necessary. For instance, a recent report showing enhanced macrophage migration upon secretion of the oxylipid prostaglandin E2 (PGE2) from pyroptotic cells [37], could be a promising avenue of investigation for AT immune recruitment mechanisms, as PGE2 is synthesised in AT [38]. This study and others also suggest that various forms of cell death, like pyroptosis or ferroptosis [39], should be scrutinised in weight‐cycled AT metaflammation.

### 2.2 Extracellular Matrix Remodelling: Role of Matrikines

An overproduction of extracellular matrix (ECM) components is a hallmark of fibrotic AT in obesity and is tightly linked to AT inflammation [40]. The steady growth and shrinkage of adipocytes during a fasting–refeeding regimen such as IF might lead to constant ECM remodeling and a potential release of chemoattractant matrikines. These matrikines are usually fragments of extracellular proteins such as collagen, noncollagen proteins and enzymes, and polysaccharides [41]. A well‐investigated matrikine is endotrophin, which is derived from proteolytic, C‐terminal cleavage of collagen type VI α3 chain, a collagen type that is abundant in obese AT [40]. Overexpression or neutralization in mice leads to an increase or a reduction of macrophage crown‐like structures and inflammatory marker expression profiles in AT, respectively [42]. Thrombospondin‐1 is another AT‐expressed, ECM‐modulating protein and in knockout mice, the HFD‐induced macrophage inflammatory phenotype was attenuated [43]. Other ECM components (like hyaluronan degradation products) and ECM‐modifying enzymes (such as matrix‐metalloproteases [MMPs] and their endogenous inhibitors TIMPs) which are associated with obesity [40], can exhibit both pro‐ and anti‐inflammatory roles [44]. These components may also contribute to an inflammatory signature that promotes the migration and polarisation of monocytes/macrophages. However, the role of adipose matrikines in a setting involving adiposity and weight cycling remains unclear.

### 2.3 Hypoxia, Adipocyte–Endothelial Interactions, and Damage‐Associated Molecular Patterns (DAMPs)

Metabolic disorders are frequently marked by compromised vascular structure and function within AT [45]. Traditionally, these vasculopathies were considered secondary to obesity and associated metabolic disorders. However, recent studies underscore the important role of endothelial cells (ECs) in regulating systemic metabolism and emphasise the bidirectional communication between the endothelium, adipocytes, and immune cells. Under conditions of chronic nutritional excess, adipocyte hypertrophy and limited angiogenic capacity induce tissue hypoxia. This low‐oxygen condition further promotes inflammation and fibrosis in AT through various mechanisms [45]. Firstly, AT hypoxia activates hypoxia‐inducible factor 1 (HIF1A) signalling in adipocytes, initiating stress response pathways that lead to adipocyte apoptosis and the release of damage‐associated molecular patterns (DAMPs) [46]. Consequently, the genetic deletion of HIF1A reduces the risk of obesity‐induced inflammation and the accumulation of macrophages and other immune cells [46]. Secondly, impairments in vascular endothelial growth factor (VEGF) signalling are a common feature in AT hypoxia [45]. Hypoxia also causes mitochondrial dysfunction, disrupting adipocyte metabolism and fostering an environment conducive to apoptosis, particularly when oxidative stress and impaired energy production ensue [47]. Consequently, weight loss and a reduction in adipocyte size should alleviate hypoxia in AT, as confirmed in previous studies [48]. However, repetitive fasting and refeeding cycles might transiently induce tissue hypoxia due to the repeated shrinkage and growth of adipocytes. This process could lead to a futile cycle of capillary rarefaction and angiogenesis, at least in the initial phase of weight loss. In support of this, IF has been shown to promote the secretion of the angiogenic protein VEGF in response to a 24‐h fast, which significantly decreased already after 6 h of refeeding [49]. Notably, prolonged fasting/refeeding cycles, which culminated in a significantly reduced body weight, enhanced VEGF‐dependent vascularization in white AT. This, in turn, stimulated macrophage polarisation to an M2 phenotype, promoting adipocyte browning, and alleviating MetSyn [49]. Furthermore, a lack of improvement in AT endothelial function following significant weight loss may limit the long‐term success of dietary interventions [12]. Consistently, the marker gene expression of capillary ECs in human AT remained unchanged after weight loss due to bariatric surgery and the estimated prevalence of these cells was indicative of successful weight maintenance [50]. Moreover, ECs communicate with adipocytes through secretory mechanisms, such as extracellular vesicles (EVs), thereby inducing adipocyte‐intrinsic signalling pathways [51]. Notably, the transfer of sphingolipid‐rich vesicles was enhanced under fasting or glucagon‐treated conditions, whereas nutrient‐rich conditions inhibited this process [51]. These data suggest that endothelial‐derived EVs play a role in the tissue response dictated by the metabolic state. However, whether this endothelial‐to‐adipocyte signalling axis affects AT inflammation has yet to be studied.

### 2.4 Monocyte Dynamics During Nutrient Fluctuation

Macrophage accumulation is not only dependent on the concentration of chemotactic signals that attract monocytes to AT but also on monocyte abundance in the blood stream and their ability to migrate into AT [52]. ECs lining the blood vessels within AT regulate this migration by expressing adhesion molecules in response to cytokines, facilitating monocyte adhesion, invasion, and extravasation to AT [52]. While long‐term fasting and weight‐loss interventions reduce adhesion molecule expression on AT ECs [53, 54], the dynamics of monocyte migration and invasion in response to repetitive fasting/feeding cycles remain unexplored. However, it has been shown that fasting alters the concentration of monocytes in the bloodstream [55, 56]. Nutrient deprivation causes monocytes to re‐enter the bone marrow and they return to the bloodstream upon refeeding. These returning monocytes are older and transcriptionally distinct, affecting their inflammatory response [55]. More recently, the accumulation of a subset of CD7^+^ monocytes in the bone marrow was connected to weight loss and regain, posing a monocyte‐centred paradigm of immune‐obesogenic memory [57]. While these CD7^+^ monocytes preferentially migrate into inguinal AT, it was not investigated whether they differentiate into macrophages. Notably, different monocyte subclasses show distinct preferences for macrophage polarisation [58], suggesting that transcriptional or epigenetic alterations in migrating monocytes might shape their trajectory towards a specific macrophage phenotype. Future studies need to explore if the dynamics of monocyte activation, migration, and invasion into AT upon weight cycling can affect long‐term weight loss. In that context, it will be essential to discriminate between the direct inflammatory or metabolic effects of monocytes and their requirement to differentiate into macrophage effector states.

### 2.5 Adipocytokines: AT‐Derived, Chemoattractant Proteins and Peptides

To consider adipocyte‐expressed proteins or peptides as chemotactic signals, messaging feeding status to circulating monocytes, they should adhere to the following criteria: (1) highly expressed in adipocytes; (2) induced by fasting or refeeding; (3) secreted as full‐length protein or proteolytically cleaved fragment and therefore found in circulation; (4) potentially acting on immune cell receptors to induce chemotaxis and/or polarisation. Many recognised adipokines harbor immuno‐modulatory properties [59]. Hence we focus on examples fulfilling the above criteria. As a corollary, angiopoietin‐like 4 (ANGPTL4) may represent a strong candidate for an adipocytokine involved in LAM recruitment under IF. ANGPTL4 is a fasting‐induced protein secreted from adipocytes and hepatocytes in mice and humans [60, 61]. It can be cleaved into an N‐ and C‐terminal fragment. The N‐terminal domain inhibits lipoprotein lipase (LPL) activity in the endothelium to restrict fatty acid import during times of energy restriction [62]. The C‐terminal domain regulates lipolysis [61, 63]. Both full‐length ANGPTL4 and its cleavage products can be detected in the circulation [61] and potentially elicit cytokine‐like signalling under fasting conditions. Moreover, the expression of the integrin ITGA5, a potential receptor for ANGPTL4, is maintained across various macrophage subclasses and monocytes. However, the level of expression differs among specific subsets [64]. In our HFD‐IF model, we performed ligand‐receptor analysis on the snRNA‐seq dataset and found that the ANGPTL4–ITGA5 pair is predicted robustly as a signalling axis between adipocytes and immune cells in the IF group, while this interaction was abrogated in case of adipocyte p53 knockout [21]. In vitro, we confirmed that Angptl4 gene expression is regulated in a nutrient‐dependent manner in cultured adipocytes and that Angptl4 may be a bona fide p53 target gene as suggested by p53 binding to the Angptl4 locus in cultured adipocytes [21]. Further studies need to evaluate the p53–ANGPTL4–ITGA5 axis as a potential monocyte/LAM recruitment mechanism upon IF.

Expression in adipocytes and circulating levels of the classical adipokines leptin, adiponectin, and resistin are waxing and waning through fasting/refeeding cycles, their receptors are expressed on macrophages and monocytes, and a spectrum of pro‐ and anti‐inflammatory actions have been reported for all three adipokines (excellently reviewed elsewhere [59]). However, using cytokine arrays in control and adipocyte‐p53 knockout (both on HFD with IF) we found no significant difference in leptin, adiponectin, or resistin plasma levels despite major differences in LAM accrual between the two groups [21]. This indicates that these classical adipokines are not directly involved in the regulation of AT LAM abundance in our model. However, indirect effects could contribute, such as the effect of adiponectin on exosomal biogenesis [65] (see below). Other adipokines, such as adipsin (CFD) and chemerin (RARRES2), have been shown to impact AT macrophage numbers, but their expression and secretion seem to be unaffected by fasting and post‐prandial states [66, 67].

When considering bona fide cytokines secreted from adipocytes in obese AT, C‐C motif chemokine ligand‐2 (CCL2 or MCP‐1) and interleukin‐6 (IL‐6) are of specific interest. CCL2 is a potent chemotactic protein and its mRNA expression was shown to increase upon glucose deprivation in cultured adipocytes [68]. Furthermore, data from whole‐body knockout mice suggested CCL2 to be necessary for macrophage recruitment to AT upon HFD [68], although another study showed no changes in a similar mouse model [69]. Plasma IL‐6 levels are increased through fasting in lean mice [70] and humans [21, 71] and it is estimated that AT‐derived IL‐6 contributes a significant proportion (∼ one‐third) to the circulating pool [59]. Importantly, adipocyte‐specific knockout of IL‐6 greatly attenuated AT macrophage accumulation under HFD [72]. However, most cytokines (like tumour necrosis factor alpha) are expressed in a broad spectrum of cells with strong expression in macrophages and many traditional methods to separate adipocytes and LAMs (like fractionation by centrifugation or flow cytometry) might not be efficient or sensitive enough to isolate and maintain either cell type into pure fractions. To date, laborious and expensive methods such as snRNA‐seq [73] might be the best options to get insight into cytokine expression in the respective cell types without cross‐contamination or destruction of fragile, lipid‐laden cells. These methods need to be combined with cell‐specific knockout models, in vivo and in vitro, to delineate the specific contribution of (adipo‐)cytokines to LAM recruitment and differentiation upon fasting/refeeding scenarios.

### 2.6 Bioactive Lipids and Other Adipose‐Relevant Metabolites

Adipocytes are highly metabolically active cells that adjust their metabolism to meet the organism's energetic demands [74]. During periods of positive energy balance and after feeding, white AT absorbs nutrients from the bloodstream and stores them as lipids. During fasting, lipids are mobilised by hydrolysing triglycerides into glycerol and fatty acids, which are then released into the circulation [74]. Transcriptomic analysis of AT from women with varying degrees of metabolic impairments and obesity revealed a strong positive association between impaired lipid metabolism and an enrichment in LAMs [75]. In line, data from our study showed that increased metabolic flexibility of adipocytes was inversely correlated with LAM abundance [21]. As a corollary, AT inflammation may be viewed as a local adaptation to primary dysfunction in adipocyte metabolism and could be triggered by metabolites released by adipocytes.

Given the capacity of AT to release lipids and the LAM marker TREM2 (triggering receptor expressed on myeloid cells 2) being a lipid receptor [76], fatty acids represent likely metabolite species that may recruit LAMs to AT and/or polarise macrophages towards the LAM phenotype in response to repetitive fasting and feeding cycles. Furthermore, in addition to TREM2, LAMs demonstrate robust expression of multiple lipid receptors such as CD36 [29]. Beyond fatty acid production, adipocytes can modulate AT remodeling by secreting other bioactive lipid products [74]. In this context, ceramides and their downstream products are potential regulators of AT inflammation in response to nutrient deprivation [77]. In adipocytes, reducing ceramide levels leads to reduced AT inflammation and alleviation of metabolic disease [78]. Furthermore, sphingolipids can directly modulate immune‐related processes, by activating inflammatory signalling cascades, and by stimulating ECs to increase immune cell adhesion and permeability [79]. Notably, the circulating levels of the sphingolipid derivate S1P were robustly increased under acute fasting conditions in lean mice [80], thereby representing a likely LAM chemoattractant. The regulation of body weight loss is highly orchestrated by a crosstalk between AT and the brain, which continuously integrates information about the body's energetic state to adjust food intake, satiety, and energy balance [81]. Hormones involved in the adipose–brain axis (such as adiponectin or leptin) play a crucial role in the regulation of appetite control and systemic energy expenditure, thereby influencing weight loss, adipose remodeling, and macrophage dynamics [81]. Signalling from central to peripheral, sympathetic nerves that innervate AT are essential in controlling adipocyte lipolysis and metabolic rate [81]. Nutrient deprivation induces catecholamine signalling, stimulating the release of norepinephrine in AT, which elevates lipolysis [74, 82]. The resulting increased lipid flux promotes the formation of crown‐like structures [83], highlighting that sympathetic signalling can function as primary mediator of AT inflammation.

In contrast to lipid metabolism, the role of adipocyte glycolysis in AT inflammation is less well documented [74]. However, during fasting and starvation, aerobic glycolysis is induced in adipocytes, which results in the enhanced production of lactate [74]. Furthermore, lactate represents a possible LAM chemoattractant, as it was reported to stimulate macrophage recruitment and to be secreted in the circulation by gut microbiota in response to every other day fasting [84]. Apart from lipid and glucose species, several other metabolites (like uridine, alpha‐ketoglutarate, uccinate, or glutamine [85]) are involved in the regulation of macrophage recruitment, but their dynamics upon fasting/refeeding cycles and properties as LAM chemoattractants have not been investigated yet.

### 2.7 Adipocyte‐Derived Extracellular Vesicles and Exosomes: Signalling Nutrient States to Immune Cells?

Apart from hormones and metabolites, interorgan crosstalk to maintain metabolic homeostasis can be achieved via secreted EVs [86]. EVs are membrane‐limited particles that carry specific cargo such as proteins, lipids, metabolites, and nucleic acids that can elicit remote effects on target cells. Exosomes are small EVs (∼100 nm) of endosomal origin and have been suggested in several studies as adipocyte‐derived signalling vehicles impacting systemic changes [65]. It was reported that AT‐secreted exosomal miRNAs signal to the liver to regulate glucose metabolism [87] and to AT‐resident macrophages, shifting their polarisation state [88]. Furthermore, exosomes secreted from adipocytes of obese mice converted bone marrow–derived macrophages towards an AT macrophage‐like phenotype [89]. Importantly, the exosome release rate from visceral adipocytes triples in adipocytes from obese as compared to lean mice and also significantly increases when mice are fasted for 16 h [89].

Together with the fact that macrophages are capable to readily internalise exosomes [90], the above studies render adipocyte‐derived exosomes in the fasting state as possible mediators of macrophage recruitment and/or differentiation. Exosomes might message via their specific cargo or via lipids contained in their membrane or cargo. However, if exosomal lipids [89], and other cargo such as miRNAs [91], reach sufficient concentrations to affect immune cell attraction under IF, needs to be scrutinised in designated studies.

### 2.8 Epigenetic Imprinting of Adipocytes and AT Macrophages

As previously discussed, weight loss does not fully restore the AT immune cell composition to the lean state in obese mouse models subjected to weight cycling [19]. This finding strongly hints towards the involvement of an immunological component in the formation of a chronic metabolic memory tied to obesity, raising the question of how such immunophenotypic imprinting is established. Obesity has been shown to significantly impact DNA methylation patterns [92], histone modifications [93], and the expression of regulatory microRNAs [94], thus rendering epigenetic mechainsms as likely key factors in the development of this immune‐obesogenic memory. In fact, a recent publication proves that humans that were formerly obese retain a distinct transcriptional signature in their AT even after weight loss. In mice, obesity‐induced epigenetic rewiring of adipocytes, which was retained after weight loss, was shown to act as a catalyst for adipocyte dysfunction and an accelerated systemic response to obesogenic signals, ultimately promoting weight regain [95]. Together with another study reporting on a lasting epigenetic memory of cold exposure in brown adipocytes [96], this presents a strong case for an essential role of the adipocyte epigenome in the formation of an AT‐intrinsic memory in response to environmental cues. However, the hypothesis that immune cells also contribute to this is supported by the fact that the epigenetic profile of macrophages can be substantially modulated by both environmental factors (e.g., nutrition [97]) and biological processes (e.g., aging [98]).

For example, it was observed that DNA methyltransferase 3b levels were increased in obesity models and after saturated fatty acid treatment of Raw264.7 macrophages, resulting in elevated DNA methylation at the Pparγ1 promoter. This, in turn, affected macrophage polarisation, which might impact the degree of metaflammation in obesity [97]. A 2021 study could further show that aged macrophages are susceptible to loss of circadian rhythmicity of gene expression. Mechanistically, the pioneer transcription factor Kruppel‐like factor 4 (KLF4) was associated with variable chromatin accessibility, distinct binding to rhythmic genes, and fluctuating expression in young but not old macrophages, likely pointing to macrophage‐specific epigenetic processes as main drivers responsible for the loss of rhythmic patterns and ultimately aggravating the age‐related decline in immune homeostasis [98].

Together, these findings suggest that epigenetic regulation of macrophages may play a crucial role in establishing a persistent obesogenic cellular memory, which may be important for long‐term weight management. Further investigations are imperative to unravel if and how obesity‐induced epigenetic reprogramming in adipocytes and immune cells could affect the outcomes of weight loss interventions.

## 3 Relevance of LAMs for Human Weight Management

### 3.1 LAMs in Human Adipose Tissue

Murine LAMs are robustly established as key modulators of AT remodelling, especially in conditions of high metabolic demand [18]. However, the role of LAMs in human AT has only been recognised recently. Consistent with the well‐documented depot‐dependent differences in AT function and cellular composition, TREM2‐positive macrophages are more prevalent in subcutaneous AT than in visceral AT in humans [75]. Given our hypothesis that LAMs reduce the sensitivity to weight loss interventions, this finding is consistent with the fact that visceral AT is more susceptible to catabolic stimuli compared to subcutaneous AT [99]. In line with data from mice, the abundance of LAMs in subcutaneous AT is strongly positively correlated with body mass index (BMI) [29, 50] and they likely enhance the inflammatory phenotype of AT by secreting various cytokines [50]. However, it remains to be determined whether the mechanisms identified in murine LAMs are conserved in humans.

### 3.2 Human Weight Loss Interventions and Inflammation

Pharmacological interventions using incretin analogues have revolutionised obesity treatment, achieving weight reductions superior to bariatric surgery or dietary interventions [9, 100]. However, surgical, lifestyle, and pharmacological weight loss methods face challenges, including lack of responsiveness and difficulty maintaining weight loss, especially upon cessation [11]. The molecular and cellular mechanisms behind these challenges are not fully understood, but sustained AT inflammation is a plausible contributor to unsuccessful long‐term weight management in humans [10].

In line, the expression of leucocyte‐specific integrins was predictive of weight maintenance in overweight and obese people [101]. During the active weight loss phase, plasma markers of low‐grade inflammation remain unchanged or even increase [102], as doe the expression of macrophage markers in AT of women on a very‐low‐calorie diet [103]. Additionally, following bariatric surgery, AT often retains its inflammatory state, as if “remembering” the obese condition despite ongoing weight loss [20]. Only significant weight loss has been shown to decrease macrophage infiltration and signs of inflammation [102, 104], suggesting that sustained inflammation may decline upon long‐term weight loss. Initial studies on AT remodelling following multi‐receptor agonist treatment have reported a reduction in AT inflammation [9]. However, future research should categorise individuals into responders and non‐responders, with respect to long‐term weight management, to examine potential differences in AT inflammation between these groups.

Theoretically, early AT inflammation during weight loss may be beneficial for tissue remodelling, while chronic inflammation might determine long‐term weight management. Our study found a more pronounced decrease in TREM2 expression in visceral AT of a follow‐up cohort two years after bariatric surgery in patients that retained more than 25% BMI loss, compared to those that regained more weight [21]. Additionally, we identified differences in responses to fasting‐mimicking diets based on genetic variants of p53 in humans [21]. These findings open the door for future studies investigating if modulating LAM abundance can improve the success of weight loss maintenance in humans.

## 4 Conclusion

Despite conceptual advances in understanding the mechanisms of LAM recruitment to AT in both obesity and in response to IF, multiple open questions remain. Some key factors have been identified. Those include fatty acids acting as signalling cues on TREM2 as a specific lipid receptor for LAMs [29], or adipocyte‐intrinsic p53 signalling possibly regulating apoptotic signals under IF [21]. However, fasting represents a highly pleiotropic stimulus, making it likely that not only one single signalling axis leads to LAM accumulation in AT, but rather that a number of signals act in concert. It is still unclear whether and how these signals operate in a coordinated manner, especially under fasting/refeeding conditions, and which signals are needed for long‐term LAM retention during weight loss. Furthermore, it is uncertain whether LAMs are exclusively recruited from circulating monocytes that adapt their polarisation state in response to the AT environment, or if monocytes or macrophages possess the ability to proliferate within the tissue itself. A particularly intriguing question is whether the abundance of LAMs in AT can serve as a predictor of weight loss efficiency and maintenance, and conversely, whether weight loss can be influenced by the abundance of LAMs in the tissue. Furthermore, it should be considered that LAMs are very plastic and can either adopt a pro‐ or anti‐inflammatory phenotypes depending on the environment, as discussed above. To address these questions, sophisticated models (e.g. in vivo lineage tracing) and methodology (e.g. snRNA‐seq [73], Zman‐seq [105], spatial transcriptomics [50]) will be necessary to track LAM dynamics upon weight cycling.

Ultimately, based on the vantage points treated in this piece (and summarised in Figure 1), dissecting which set of signals recruit monocytes, which leads to polarisation into LAMs, and which orchestrates LAM retention, will be important to reveal therapeutic opportunities that inform long‐term weight management strategies based on modulating LAM abundance in AT.

## Author Contributions

The three authors contributed equal parts to the manuscript. Final editing was done by Andreas Prokesch. All authors approved the final manuscript.

## Conflicts of Interest

The authors declare that the article was written without any commercial or financial relationships that could be construed as a potential conflict of interest.

## Data Availability

Data sharing does not apply to this article as no datasets were generated or analysed during the current study.
